# 4-{[4-(Dimethylamino)benzylidene]­amino}-1,5-dimethyl-2-phenyl-1*H*-pyrazol-3(2*H*)-one

**DOI:** 10.1107/S1600536810023536

**Published:** 2010-06-23

**Authors:** Abdullah M. Asiri, Salman A. Khan, Kong Wai Tan, Seik Weng Ng

**Affiliations:** a Chemistry Department, Faculty of Science, King Abdul Aziz University, PO Box 80203, Jeddah 21589, Saudi Arabia; b Department of Chemistry, University of Malaya, 50603 Kuala Lumpur, Malaysia

## Abstract

The azomethine double-bond in the title Schiff base, C_20_H_22_N_4_O, has an *E*-configuration. The aromatic ring of the benzyl­idene portion (r.m.s. deviation 0.011 Å) and the five-membered pyrazolyl ring (r.m.s. deviation 0.033 Å) form a dihedral angle of 19.0 (1)°. The phenyl substituent is twisted by 55.0 (1)° with respect to the five-membered ring.

## Related literature

For background to Schiff bases derived from 4-amino­anti­pyridine, see: Montalvo-González & Ariza-Castolo (2003 ▶).
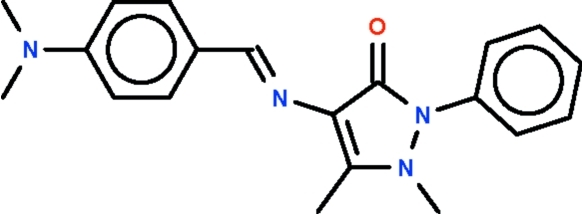


## Experimental

### 

#### Crystal data

C_20_H_22_N_4_O
*M* *_r_* = 334.42
Monoclinic, 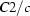

*a* = 17.7275 (14) Å
*b* = 6.7552 (6) Å
*c* = 29.387 (2) Å
β = 101.426 (1)°
*V* = 3449.5 (5) Å^3^
*Z* = 8
Mo *K*α radiation
μ = 0.08 mm^−1^
*T* = 100 K
0.25 × 0.20 × 0.10 mm

#### Data collection

Bruker SMART APEX diffractometer
15916 measured reflections
3959 independent reflections
3146 reflections with *I* > 2σ(*I*)
*R*_int_ = 0.043

#### Refinement

*R*[*F* ^2^ > 2σ(*F* ^2^)] = 0.041
*wR*(*F* ^2^) = 0.101
*S* = 1.02
3959 reflections
230 parameters
H-atom parameters constrained
Δρ_max_ = 0.22 e Å^−3^
Δρ_min_ = −0.22 e Å^−3^

### 

Data collection: *APEX2* (Bruker, 2009 ▶); cell refinement: *SAINT* (Bruker, 2009 ▶); data reduction: *SAINT*; program(s) used to solve structure: *SHELXS97* (Sheldrick, 2008 ▶); program(s) used to refine structure: *SHELXL97* (Sheldrick, 2008 ▶); molecular graphics: *X-SEED* (Barbour, 2001 ▶); software used to prepare material for publication: *publCIF* (Westrip, 2010 ▶).

## Supplementary Material

Crystal structure: contains datablocks global, I. DOI: 10.1107/S1600536810023536/kp2268sup1.cif

Structure factors: contains datablocks I. DOI: 10.1107/S1600536810023536/kp2268Isup2.hkl

Additional supplementary materials:  crystallographic information; 3D view; checkCIF report

## Figures and Tables

**Fig. 1.**
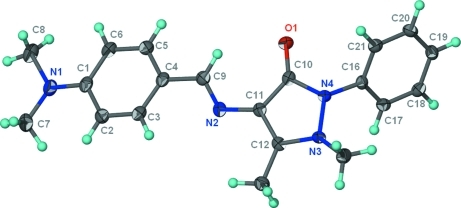
ORTEP drawing (Barbour, 2001) of the title molecule (I) with the displacement parameters at the 70% probability level; hydrogen atoms are drawn as spheres of arbitrary radius.

## supplementary crystallographic information

### Comment

4-Aminoantipyrine (4-amino-1,2-dihydro-1,5-dimethyl-2-phenyl-3*H*-pyrazol-3-one) possesses a aminopyrazolone unit, a feature that allows the compound to condense with aromatic aldehydes to yield Schiff bases. The Schiff base derived from the benzaldehyde homolog has nearly coplanar phenyl and pyrazoly rings (Montalvo-González & Ariza-Castolo, 2003). The azomethine double-bond in the Schiff base, C_20_H_22_N_4_O, has an *E*-configuration (Scheme 1, Fig. 1). The aromatic ring of the benzylidene portion (r.m.s. deviation 0.011 Å) and 5-membered pyrazolyl ring (r.m.s. deviation 0.033 Å) form the dihedral angle between of 19.0 (1) °. The phenyl substituent is twisted by 55.0 (1) ° with respect to the 5-membered ring.

### Experimental

*N*,*N*-Dimethylbenzaldehyde (0.32 g, 2.2 mmol) and 4-aminoantipyrine (0.45 g, 2.2 mmol) were heated in methanol (15 ml) for 5 h. A solution was set aside to cool slowly and after a day crystals were separated.

### Refinement

Carbon-bound H-atoms were placed in calculated positions [C–H 0.95 to 0.98 Å, *U*(H) 1.2 to 1.5*U*_eq_(C)] and were included in the refinement in the riding model approximation.

### Crystal data

**Table:**

C_20_H_22_N_4_O	*F*(000) = 1424
*M**_r_* = 334.42	*D*_x_ = 1.288 Mg m^−^^3^
Monoclinic, *C*2/*c*	Mo *K*α radiation, λ = 0.71073 Å
Hall symbol: -C 2yc	Cell parameters from 3746 reflections
*a* = 17.7275 (14) Å	θ = 2.3–28.2°
*b* = 6.7552 (6) Å	µ = 0.08 mm^−^^1^
*c* = 29.387 (2) Å	*T* = 100 K
β = 101.426 (1)°	Irregular, yellow
*V* = 3449.5 (5) Å^3^	0.25 × 0.20 × 0.10 mm
*Z* = 8	

### Data collection

**Table:**

Bruker SMART APEX diffractometer	3146 reflections with *I* > 2σ(*I*)
Radiation source: fine-focus sealed tube	*R*_int_ = 0.043
graphite	θ_max_ = 27.5°, θ_min_ = 1.4°
ω scans	*h* = −22→22
15916 measured reflections	*k* = −8→8
3959 independent reflections	*l* = −38→38

### Refinement

**Table:**

Refinement on *F*^2^	Primary atom site location: structure-invariant direct methods
Least-squares matrix: full	Secondary atom site location: difference Fourier map
*R*[*F*^2^ > 2σ(*F*^2^)] = 0.041	Hydrogen site location: inferred from neighbouring sites
*wR*(*F*^2^) = 0.101	H-atom parameters constrained
*S* = 1.02	*w* = 1/[σ^2^(*F*_o_^2^) + (0.0456*P*)^2^ + 1.6458*P*] where *P* = (*F*_o_^2^ + 2*F*_c_^2^)/3
3959 reflections	(Δ/σ)_max_ = 0.001
230 parameters	Δρ_max_ = 0.22 e Å^−^^3^
0 restraints	Δρ_min_ = −0.22 e Å^−^^3^

### Fractional atomic coordinates and isotropic or equivalent isotropic displacement parameters (Å^2^)

**Table:**

	*x*	*y*	*z*	*U*_iso_*/*U*_eq_	
O1	0.44182 (5)	0.85565 (14)	0.62281 (3)	0.0206 (2)	
N1	0.88210 (7)	0.91371 (18)	0.54534 (4)	0.0232 (3)	
N2	0.58578 (6)	0.58777 (17)	0.61620 (4)	0.0173 (2)	
N3	0.45341 (6)	0.41420 (16)	0.68429 (4)	0.0163 (2)	
N4	0.41440 (6)	0.58791 (16)	0.66631 (4)	0.0166 (2)	
C1	0.81084 (8)	0.8721 (2)	0.55470 (4)	0.0196 (3)	
C2	0.79273 (8)	0.6806 (2)	0.56883 (4)	0.0204 (3)	
H2	0.8292	0.5767	0.5700	0.024*	
C3	0.72303 (8)	0.6430 (2)	0.58096 (4)	0.0194 (3)	
H3	0.7125	0.5130	0.5903	0.023*	
C4	0.66719 (8)	0.7900 (2)	0.57993 (4)	0.0182 (3)	
C5	0.68396 (8)	0.9775 (2)	0.56453 (5)	0.0210 (3)	
H5	0.6466	1.0795	0.5626	0.025*	
C6	0.75351 (8)	1.0189 (2)	0.55193 (5)	0.0219 (3)	
H6	0.7627	1.1477	0.5413	0.026*	
C7	0.93806 (8)	0.7568 (2)	0.54517 (5)	0.0253 (3)	
H7A	0.9402	0.6730	0.5726	0.038*	
H7B	0.9889	0.8150	0.5457	0.038*	
H7C	0.9229	0.6766	0.5171	0.038*	
C8	0.89519 (9)	1.0978 (2)	0.52251 (5)	0.0267 (3)	
H8A	0.8791	1.2096	0.5396	0.040*	
H8B	0.8653	1.0975	0.4907	0.040*	
H8C	0.9500	1.1105	0.5219	0.040*	
C9	0.59614 (8)	0.7527 (2)	0.59633 (4)	0.0183 (3)	
H9	0.5571	0.8510	0.5923	0.022*	
C10	0.45790 (7)	0.68849 (19)	0.63871 (4)	0.0162 (3)	
C11	0.52186 (8)	0.55737 (19)	0.63643 (4)	0.0160 (3)	
C12	0.51466 (7)	0.39349 (19)	0.66245 (4)	0.0160 (3)	
C13	0.56351 (8)	0.2136 (2)	0.66934 (5)	0.0206 (3)	
H13A	0.6052	0.2271	0.6521	0.031*	
H13B	0.5322	0.0975	0.6580	0.031*	
H13C	0.5854	0.1973	0.7025	0.031*	
C14	0.40306 (8)	0.2478 (2)	0.69032 (5)	0.0208 (3)	
H14A	0.4343	0.1376	0.7054	0.031*	
H14B	0.3744	0.2051	0.6599	0.031*	
H14C	0.3668	0.2897	0.7097	0.031*	
C16	0.36949 (7)	0.68935 (18)	0.69429 (4)	0.0154 (3)	
C17	0.39115 (7)	0.68983 (19)	0.74243 (4)	0.0168 (3)	
H17	0.4349	0.6172	0.7574	0.020*	
C18	0.34793 (8)	0.79786 (19)	0.76822 (5)	0.0181 (3)	
H18	0.3619	0.7977	0.8011	0.022*	
C19	0.28464 (8)	0.90595 (19)	0.74637 (5)	0.0187 (3)	
H19	0.2559	0.9817	0.7642	0.022*	
C20	0.26332 (8)	0.9032 (2)	0.69835 (5)	0.0196 (3)	
H20	0.2198	0.9770	0.6833	0.024*	
C21	0.30522 (8)	0.7932 (2)	0.67215 (5)	0.0180 (3)	
H21	0.2900	0.7890	0.6393	0.022*	

### Atomic displacement parameters (Å^2^)

**Table:**

	*U*^11^	*U*^22^	*U*^33^	*U*^12^	*U*^13^	*U*^23^
O1	0.0237 (5)	0.0167 (5)	0.0222 (5)	0.0029 (4)	0.0069 (4)	0.0043 (4)
N1	0.0196 (6)	0.0284 (7)	0.0230 (6)	−0.0015 (5)	0.0074 (5)	0.0046 (5)
N2	0.0160 (6)	0.0207 (6)	0.0153 (5)	−0.0014 (5)	0.0029 (4)	−0.0016 (4)
N3	0.0177 (6)	0.0124 (5)	0.0193 (5)	0.0016 (4)	0.0047 (5)	0.0016 (4)
N4	0.0183 (6)	0.0141 (5)	0.0180 (5)	0.0028 (4)	0.0053 (5)	0.0028 (4)
C1	0.0196 (7)	0.0273 (7)	0.0117 (6)	−0.0017 (6)	0.0027 (5)	0.0007 (5)
C2	0.0198 (7)	0.0249 (7)	0.0166 (6)	0.0024 (6)	0.0038 (5)	0.0014 (5)
C3	0.0217 (7)	0.0213 (7)	0.0152 (6)	−0.0014 (6)	0.0034 (5)	0.0018 (5)
C4	0.0192 (7)	0.0229 (7)	0.0123 (6)	−0.0011 (6)	0.0029 (5)	0.0006 (5)
C5	0.0219 (7)	0.0233 (7)	0.0178 (6)	0.0031 (6)	0.0042 (6)	0.0026 (5)
C6	0.0249 (8)	0.0220 (7)	0.0193 (7)	−0.0031 (6)	0.0060 (6)	0.0034 (6)
C7	0.0196 (7)	0.0347 (8)	0.0226 (7)	−0.0002 (6)	0.0067 (6)	0.0001 (6)
C8	0.0270 (8)	0.0313 (8)	0.0229 (7)	−0.0074 (7)	0.0079 (6)	0.0021 (6)
C9	0.0198 (7)	0.0206 (7)	0.0141 (6)	0.0005 (5)	0.0024 (5)	−0.0009 (5)
C10	0.0167 (7)	0.0173 (7)	0.0145 (6)	−0.0032 (5)	0.0026 (5)	−0.0016 (5)
C11	0.0161 (7)	0.0178 (7)	0.0138 (6)	−0.0004 (5)	0.0023 (5)	−0.0020 (5)
C12	0.0152 (7)	0.0166 (6)	0.0150 (6)	−0.0008 (5)	0.0005 (5)	−0.0036 (5)
C13	0.0211 (7)	0.0184 (7)	0.0224 (7)	0.0016 (6)	0.0044 (6)	0.0000 (5)
C14	0.0232 (7)	0.0164 (7)	0.0238 (7)	−0.0026 (5)	0.0072 (6)	0.0008 (5)
C16	0.0157 (6)	0.0128 (6)	0.0191 (6)	−0.0022 (5)	0.0066 (5)	−0.0012 (5)
C17	0.0145 (7)	0.0155 (6)	0.0196 (6)	−0.0006 (5)	0.0017 (5)	0.0018 (5)
C18	0.0207 (7)	0.0168 (7)	0.0168 (6)	−0.0032 (5)	0.0039 (5)	0.0006 (5)
C19	0.0193 (7)	0.0147 (6)	0.0242 (7)	0.0000 (5)	0.0095 (6)	−0.0009 (5)
C20	0.0161 (7)	0.0166 (6)	0.0258 (7)	0.0019 (5)	0.0036 (6)	0.0032 (5)
C21	0.0179 (7)	0.0183 (7)	0.0176 (6)	−0.0009 (5)	0.0025 (5)	0.0019 (5)

### Geometric parameters (Å, °)

**Table:**

O1—C10	1.2337 (16)	C8—H8A	0.9800
N1—C1	1.3743 (18)	C8—H8B	0.9800
N1—C7	1.4523 (19)	C8—H8C	0.9800
N1—C8	1.4533 (19)	C9—H9	0.9500
N2—C9	1.2877 (17)	C10—C11	1.4509 (18)
N2—C11	1.3950 (17)	C11—C12	1.3657 (18)
N3—C12	1.3735 (17)	C12—C13	1.4825 (18)
N3—N4	1.4106 (15)	C13—H13A	0.9800
N3—C14	1.4676 (17)	C13—H13B	0.9800
N4—C10	1.4007 (16)	C13—H13C	0.9800
N4—C16	1.4279 (16)	C14—H14A	0.9800
C1—C6	1.410 (2)	C14—H14B	0.9800
C1—C2	1.415 (2)	C14—H14C	0.9800
C2—C3	1.3758 (19)	C16—C21	1.3855 (18)
C2—H2	0.9500	C16—C17	1.3905 (18)
C3—C4	1.3982 (19)	C17—C18	1.3872 (18)
C3—H3	0.9500	C17—H17	0.9500
C4—C5	1.3969 (19)	C18—C19	1.3853 (19)
C4—C9	1.4569 (19)	C18—H18	0.9500
C5—C6	1.3842 (19)	C19—C20	1.3867 (19)
C5—H5	0.9500	C19—H19	0.9500
C6—H6	0.9500	C20—C21	1.3871 (19)
C7—H7A	0.9800	C20—H20	0.9500
C7—H7B	0.9800	C21—H21	0.9500
C7—H7C	0.9800		
			
C1—N1—C7	120.48 (12)	N2—C9—H9	119.7
C1—N1—C8	120.29 (12)	C4—C9—H9	119.7
C7—N1—C8	116.83 (12)	O1—C10—N4	123.44 (12)
C9—N2—C11	121.43 (12)	O1—C10—C11	131.70 (12)
C12—N3—N4	106.50 (10)	N4—C10—C11	104.81 (11)
C12—N3—C14	122.31 (11)	C12—C11—N2	122.13 (12)
N4—N3—C14	114.67 (10)	C12—C11—C10	107.96 (11)
C10—N4—N3	109.57 (10)	N2—C11—C10	129.65 (12)
C10—N4—C16	122.30 (11)	C11—C12—N3	110.45 (11)
N3—N4—C16	118.13 (10)	C11—C12—C13	128.60 (12)
N1—C1—C6	121.69 (13)	N3—C12—C13	120.93 (12)
N1—C1—C2	121.11 (13)	C12—C13—H13A	109.5
C6—C1—C2	117.18 (12)	C12—C13—H13B	109.5
C3—C2—C1	120.83 (13)	H13A—C13—H13B	109.5
C3—C2—H2	119.6	C12—C13—H13C	109.5
C1—C2—H2	119.6	H13A—C13—H13C	109.5
C2—C3—C4	122.11 (13)	H13B—C13—H13C	109.5
C2—C3—H3	118.9	N3—C14—H14A	109.5
C4—C3—H3	118.9	N3—C14—H14B	109.5
C5—C4—C3	117.13 (12)	H14A—C14—H14B	109.5
C5—C4—C9	121.16 (12)	N3—C14—H14C	109.5
C3—C4—C9	121.64 (12)	H14A—C14—H14C	109.5
C6—C5—C4	121.84 (13)	H14B—C14—H14C	109.5
C6—C5—H5	119.1	C21—C16—C17	120.82 (12)
C4—C5—H5	119.1	C21—C16—N4	118.22 (11)
C5—C6—C1	120.84 (13)	C17—C16—N4	120.92 (12)
C5—C6—H6	119.6	C18—C17—C16	119.08 (12)
C1—C6—H6	119.6	C18—C17—H17	120.5
N1—C7—H7A	109.5	C16—C17—H17	120.5
N1—C7—H7B	109.5	C19—C18—C17	120.55 (12)
H7A—C7—H7B	109.5	C19—C18—H18	119.7
N1—C7—H7C	109.5	C17—C18—H18	119.7
H7A—C7—H7C	109.5	C18—C19—C20	119.82 (12)
H7B—C7—H7C	109.5	C18—C19—H19	120.1
N1—C8—H8A	109.5	C20—C19—H19	120.1
N1—C8—H8B	109.5	C19—C20—C21	120.27 (13)
H8A—C8—H8B	109.5	C19—C20—H20	119.9
N1—C8—H8C	109.5	C21—C20—H20	119.9
H8A—C8—H8C	109.5	C16—C21—C20	119.44 (12)
H8B—C8—H8C	109.5	C16—C21—H21	120.3
N2—C9—C4	120.66 (13)	C20—C21—H21	120.3
			
C12—N3—N4—C10	−8.61 (13)	C9—N2—C11—C10	1.4 (2)
C14—N3—N4—C10	−147.15 (11)	O1—C10—C11—C12	176.26 (14)
C12—N3—N4—C16	−155.05 (11)	N4—C10—C11—C12	−1.17 (14)
C14—N3—N4—C16	66.41 (14)	O1—C10—C11—N2	2.2 (2)
C7—N1—C1—C6	−175.50 (12)	N4—C10—C11—N2	−175.21 (12)
C8—N1—C1—C6	−13.63 (19)	N2—C11—C12—N3	170.36 (11)
C7—N1—C1—C2	6.28 (19)	C10—C11—C12—N3	−4.23 (15)
C8—N1—C1—C2	168.15 (13)	N2—C11—C12—C13	−8.4 (2)
N1—C1—C2—C3	175.89 (12)	C10—C11—C12—C13	177.05 (12)
C6—C1—C2—C3	−2.41 (19)	N4—N3—C12—C11	7.87 (14)
C1—C2—C3—C4	0.1 (2)	C14—N3—C12—C11	142.49 (12)
C2—C3—C4—C5	1.99 (19)	N4—N3—C12—C13	−173.29 (11)
C2—C3—C4—C9	−175.04 (12)	C14—N3—C12—C13	−38.67 (18)
C3—C4—C5—C6	−1.7 (2)	C10—N4—C16—C21	68.38 (16)
C9—C4—C5—C6	175.37 (12)	N3—N4—C16—C21	−149.66 (12)
C4—C5—C6—C1	−0.7 (2)	C10—N4—C16—C17	−109.37 (14)
N1—C1—C6—C5	−175.58 (12)	N3—N4—C16—C17	32.58 (17)
C2—C1—C6—C5	2.7 (2)	C21—C16—C17—C18	−0.70 (19)
C11—N2—C9—C4	173.20 (12)	N4—C16—C17—C18	177.00 (11)
C5—C4—C9—N2	−170.60 (12)	C16—C17—C18—C19	−0.84 (19)
C3—C4—C9—N2	6.3 (2)	C17—C18—C19—C20	1.3 (2)
N3—N4—C10—O1	−171.71 (12)	C18—C19—C20—C21	−0.2 (2)
C16—N4—C10—O1	−26.94 (19)	C17—C16—C21—C20	1.8 (2)
N3—N4—C10—C11	5.99 (13)	N4—C16—C21—C20	−175.97 (12)
C16—N4—C10—C11	150.76 (11)	C19—C20—C21—C16	−1.3 (2)
C9—N2—C11—C12	−171.88 (12)		

## References

[bb1] Barbour, L. J. (2001). *J. Supramol. Chem.* **1**, 189–191.

[bb2] Bruker (2009). *APEX2* and *SAINT* Bruker AXS Inc., Madison, Wisconsin, USA.

[bb3] Montalvo-González, R. & Ariza-Castolo, A. (2003). *J. Mol. Struct.* **655**, 375–389.

[bb4] Sheldrick, G. M. (2008). *Acta Cryst.* A**64**, 112–122. 10.1107/S0108767307043930 18156677

[bb5] Westrip, S. P. (2010). *J. Appl. Cryst.* **43** Submitted.

